# From brewery waste to agricultural wealth: Enhancing nitrogen use efficiency and productivity of maize through brewery sludge and blended NPS fertilizer in North Mecha District, Northwestern Ethiopia

**DOI:** 10.1371/journal.pone.0319958

**Published:** 2025-05-08

**Authors:** Fenta Assefa, Yigrem Mengist, Habatamu Yigermal, Kelemu Nakachew

**Affiliations:** 1 Department of Plant Sciences, College of Agriculture and Environmental Sciences, University of Gondar, Gondar, Ethiopia; 2 Department of Horticulture, Burie Campus, Debre Markos University, Ethiopia; 3 Department of Plant Science, College of Agriculture and Natural Resource Management, Debre Markos University, Debre Markos, Ethiopia; University of Minnesota, UNITED STATES OF AMERICA

## Abstract

The expansion of brewery factories with huge production potential of brewery sludge in Ethiopia presents a significant opportunity to enhance sustainable soil and crop productivity. Hence, this field experiment was conducted in North Mecha district, Northwestern Ethiopia, to improve the yield and nnitrogen use efficiency of maize by applying brewery sludge (BS), blended nitrogen, phosphorous and sulfur (NPS) fertilizers alone and in combination. Six treatments (T1: control, T2: 100% recommended dose of blended nitrogen, phosphorous, sulfur (RDNPS (100 kg NPS ha^-1^)); T3: 75% RDNPS +  25% recommended dose of brewery sludge (RDBS); T4: 50% RDNPS +  50% RDBS; T5: 25% RDNPS +  75% RDBS; T6: 100% RDBS (10 t BS ha^-1^) were laid out in a Randomized Complete Block Design (RCBD) with three replications. The results revealed that both single and combined fertilizer applications resulted in higher production, nitrogen uptake, and efficiency as compared to no fertilizer application. Notably, the combined application of 75% RDBS with 25% RDNPS produced the highest above-ground biomass yield (23161.9 kg ha^-1^), grain yield (10620.6 kg ha^-1^), stover yield (12541.3 kg ha^-1^), harvest index (45.85%), nitrogen concentration in grain (1.71%) and stover (1.00%), as well as grain (181.72 kg ha^-1^), stover (124.17 kg ha^-1^), and total (305.89 kg ha^-1^) nitrogen uptake. Furthermore, the combined application of 75% RDBS with 25% RDNPS produced the highest grain yield (10620.6 kg ha^-1^), net benefit (170987.97 Ethiopian Birr (ETB) ha^-1^), and an acceptable marginal rate of return (MRR) (12613.93%) for maize production in the region. Hence, the study reveals that using BS and blended NPS at precise ratios can improve maize productivity in the North Mecha district. However, as the experiment was carried out only in one location for one cropping season, further studies at different locations for several years or seasons should be conducted to come up with strong and reliable recommendations.

## Introduction

Maize (*Zea mays*) is one of the major cereal crops grown in Ethiopia with high yield potentials of 15.8 t ha^-1^ compared to other major cereal crops grown in the country [1]. However, its current national average yield is less than four t ha^-1^ due to several production constraints [2–5]. The most significant limiting factors affecting its productivity were declining soil fertility and low nutrient use efficiency [5–9], poor agronomic practices [10], limited use of inputs, poor seed quality, disease and insect pests [11].

Among the aforementioned constraints, declining soil fertility is the major biophysical and manageable abiotic stress limiting maize productivity and food self-sufficiency under increasing population pressure [5,7,12–15]. Unbalanced and continuous application of limited chemical fertilizers such as Diammonium phosphate (DAP: 18% N and 46% P_2_O_5_) and Urea (46% N) over an extended period is a primary factor contributing to the decline in soil fertility which leads to low nutrient (N) use efficiency, nutrient response, and maize performance in Ethiopia [16]. This practice, both in terms of quantity and type, can worsen the depletion of essential macronutrients like K, Mg, Ca, S and micronutrients like B, Zn, Cu that are not adequately provided by chemical fertilizers and thereby lead to chemical soil degradation [17–23]. Due to the impact of imbalanced nutrient supply on plant nutrient uptake and utilization, crop yield is reduced [16]. Therefore, it is crucial to apply the right nutrient source at the recommended rate, at the right time during the growing season, and with the right application methods (referred to as the 4R’s) to optimize crop nutrient use efficiency [24,25]. However, 30–40% of Ethiopian farmers often find it expensive to use the recommended rates, leading them to apply lower recommended rates (37–40 kg ha^-1^ (NPS +  Urea)) [26], which resulted in low nutrient use efficiency and crop yield. Consequently, addressing this issue has become a top priority and a significant national concern, prompting various initiatives to counteract the adverse effects of chemical farming. Because continual application of chemical fertilizers can lead to changes in soil pH, acidification, and soil compaction, ultimately reducing organic and humus content and beneficial organisms, increasing leaching of nutrients (N), hindering plant growth, and potentially contributing to greenhouse gas emissions [27]. As a result, the utilization of organic fertilizers has emerged as a viable solution for integrating nutrient supply systems in agriculture to efficiently utilize applied nutrients and increase crop productivity [28].

Hence, in Ethiopia, there is an expansion of brewery factories with the production potential of substantial amounts of sludge, which can be utilized as an organic soil amendment for sustainable crop production. As the sludge is also an environmentally friendly, renewable source of nutrients for crops with almost no toxic effects [29,30], it can be directly applied to soil to amend and restore its fertility [31]. It is also more effective than traditional organic fertilizer, as it contains 20–30% more nutrients than commonly used organic fertilizer [32] and reduces the application of chemical fertilizers by 40–50% [29]. Studies have demonstrated that the application of BS can significantly increase crop yield, improve soil fertility by enhancing physical and chemical properties of soil, beneficial microorganisms and serve as a valuable source of nutrients like nitrogen, phosphorus, potassium and other micronutrients [33–35]. Moreover, in Ethiopia, there is limited research on the utilization of brewery sludge as an organic soil amendment. Nonetheless, its application to agricultural land has been found to have a positive impact on soil pH, electrical conductivity, and the overall productivity of crops like sorghum [36], haricot bean [37], wheat [35], potato [38], and maize [39] compared to chemical fertilizers. However, its sole application is also limited by a slow release of nutrients, low nutrient content and high labor demand for preparation and transportation [40]. Thus, soil fertility can be effectively restored and crop productivity enhanced by implementing the concept of integrated soil fertility management [41]. The combined use of organic and inorganic nutrient sources also helps to maintain high nutrient use efficiency, soil and crop productivity [42,43]. In light of this, an important area of research in the North Mecha district was the use of BS in combination with chemical fertilizers to enhance nitrogen use efficiency and maize production sustainably.

## Materials and methods

### Description of the study area

A field experiment was conducted during the main cropping season from June to December 2021 in Kudmi village, found within the North Mecha district, at the research and technology demonstration and transfer site of the College of Agriculture and Environmental Sciences, Bahir Dar University. The topography of the experimental area is a gentle slope, and the area is located at 11° 20′ N to 11° 25′ N latitude and 37° 05′ E to 37° 09′ E longitude with an altitude of 1960 m above sea level [44]. According to the Bahir Dar Meteorology Station report (unpublished), the area receives an annual mean rainfall of approximately 1395 mm. The daily mean minimum and maximum temperatures of the area are 12.8 and 27°C, respectively. Crop-livestock mixed farming is the dominant production system in the district. The main crops cultivated are maize, finger millet, teff, barley, pulses and oil crops.

### Description of the experimental material

The Bako Hybrid (BH)-661 maize (*Zea mays*) variety was used as a test crop in the study as previously used in the same research by Assefa *et al* [45].The BH-661 variety has high productivity (average 12 t ha^-1^) and extensive average coverage in Ethiopia, particularly in the Amhara region [46]. As shown below in Table 1, Blended NPS fertilizer containing 19% N, 38% P_2_O_5_ (16.59% P), 7% S and BS that used malt barely as a raw material for beer production, containing 4.23% N, 0.176% P, 0.377 mg/g Zn, 0.0043 mg/g Cd, 0.091 mg/g Cr, 0.054 mg/g Cu and 0.017 mg/g Ni [49] collected from the Dashen brewery company located at Gondar was used as the experimental material.

**Table 1:** Treatment combinations and nutrient content for maize productivity as influenced by soil amendments.

Treatments	Applied Nutrients (kg ha^-1^) from fertilizer sources							
	Nitrogen (N)	Phosphorus (P)	Sulfur (S) *	Zinc (Zn)	Cadmium (Cd)	Chromium (Cr)	Copper (Cu)	Nickel (Ni)
Control	0	0	0	0	0	0	0	0
100% RDNPS	19	38	7	0	0	0	0	0
75% RDNPS + 25% RDBS	120	32.9	5.25	0.94	0.011	0.23	0.14	0.043
50% RDNPS + 50% RDBS	221	27.8	3.5	1.89	0.022	0.46	0.27	0.085
25% RDNPS + 75% RDBS	322	22.7	1.75	2.83	0.033	0.68	0.41	0.13
100% RDBS	423	17.6	Nd	3.77	0.043	0.91	0.54	0.17

•= the sulfur content is only from blended NPS fertilizer; **RDNPS** =  recommended dose of blended NPS fertilizer (100 kg ha^-1^) for maize in the district; **RDBS** =  recommended dose of brewery sludge (10 t ha^-1^) for maize production in the district; **Nd** =  not determined.

Note: Limit for Zn =  7 kg, Cd =  0.03 kg, Cr =  2.1 kg, Cu =  4 kg, Ni =  0.62 kg in 10 tone of brewery sludge, respectively [47].

Limit for Zn =  75 kg, Cd =  0.85 kg, Cr =  30 kg, Cu =  43 kg, Ni =  4.2 kg in 10 tone of brewery sludge, respectively [48].

### Experimental treatments, design and procedures

Six treatments were imposed in the experiment, which was carried out using a randomized complete block design (RCBD) with three replications. Standard recommendation rates were followed for fertilizer rates, 100 kg blended NPS ha^-1^ fertilizer and 10 t BS ha^-1^ [45]. The treatments were T1: control, T2: 100% RDNPS; T3: 75% RDNPS +  25% RDBS; T4: 50% RDNPS +  50% RDBS; T5: 25% RDNPS +  75% RDBS; T6: 100% RDBS (Table 1). The land was prepared with oxen/animal-drawn plowing and hand tools and subsequently leveled and smoothed by human labor using hand tools. As per the design and treatments, the experimental field was then manually subdivided into blocks and plots based on the design and treatments. Then, BS was applied three weeks before sowing to favor the nutrient break down and blended NPS fertilizer was applied during sowing to ready for crops at a depth of 20 cm. The gross land area of the experiment was 13.25 m × 23.5 m (311.375 m^2^) and a gross plot size of 3.75 m ×  3 m (11.25 m^2^). A 1.0 m wide-open path separated the adjacent blocks, and a 0.5 m wide path separated plots within a block from one another. Using a random table number, the experimental treatments were assigned to the experimental plots in each block. As a result, there were five rows totaling 3 m in length, with 10 plants per row and 50 plants per plot. To avoid possible border effects, the net plot size (harvestable area) was 2.25 m ×  2.4 m (5.4 m^2^) (i.e., the middle 3 rows from each plot) by excluding the guard rows of each plot horizontally and a 0.3 m row segment from both ends of the plot vertically. All the remaining necessary agronomic practices and crop management activities were subsequently performed uniformly.

The study involved standard agricultural practices and did not include any invasive methods or the collection of all the data on maize, no additional permits were required from governmental or regulatory bodies.

### Data collection and measurements

#### Soil data.

Prior to sowing, soil samples were taken from the entire experimental field at a depth of 0–20 cm in a diagonal pattern using a soil auger following plowing. The samples were combined and thoroughly mixed to create a composite sample weighing one kilogram, which was then air-dried and ground to pass through a 2 mm sieve for analysis of specific soil physicochemical properties, including particle size distribution, soil pH, total nitrogen, available phosphorous and cation exchange capacity (CEC). Particle distribution in the soil was assessed using the hydrometer method as developed by Ref [50]. Soil pH was measured potentiometrically in a 1:2.5 soil-water suspension as described in Ref [51]. Total nitrogen was assessed via the micro-Kjeldahl method as Ref [52], organic carbon via the wet digestion method as outlined in Ref [53], and available phosphorus following Olsen [54]. CEC was determined from NH_4_OAc-saturated samples using the micro-Kjeldahl procedure [55].

#### Crop data.

Aboveground dry biomass yield (AGDBY) was determined by weighing the whole aboveground plant parts from the net plot area after complete oven-drying at 105˚C for 24 h. Grain yield was determined from the net harvestable area sampled for AGDBY determination; the yield was adjusted to 12.5% moisture content, and the result converted to kg ha^-1^.


$$Grain\;yield\left(kg\;ha^{-1}\right)\;at\;12.5\%\;moisture\;base=yield\;obtained\;\left(kg\;ha^{-1}\right)\times \frac{100-\text{\%MC}}{100-12.5},$$ (1)

where MC = grain moisture content. Stover yield was measured by taking the weight of the straw harvested from the net plot area of each plot and converting it to kilograms per hectare after sun drying the straw. Harvest index (HI): The HI was calculated as the ratio of grain yield to total aboveground dry biomass yield multiplied by 100 at harvest from the indicated treatments [56].


$$HI\left(\%\right)=\frac{\text{Grain yield}}{\text{Aboveground dry biomass yield }}\times 100$$ (2)

*For plant tissue analysis at maturity,* representative non-border maize plant samples were randomly collected from each plot, separated into grain and straw, and stored in paper bags. The grain and straw samples from each treatment were oven-dried at 70°C to a constant weight, ground using a stainless steel grinder, re-dried at 60°C, and ashed in a muffle furnace at 550°C for eight hours. The dried samples were milled, and the N contents of the grain and straw samples were determined using the micro-Kjeldahl method as outlined by the American Association of Cereal Chemists (AACC) [57].

*The following N-efficiency parameters were calculated for each treatment following* [58]:

1. *Nitrogen uptake* was calculated by multiplying the grain and straw yield (kg ha^ − 1^) by the nitrogen concentration (%) of each treatment as follows:

$$Nitrogen\;uptake\;by\;grain\;or\;straw\left(kg\;ha^{-1}\right)=\left[yield\;of\;grain\;or\;straw\left(kg\;ha^{-1}\right)\times nitrogen\;concentration\;of\;grain\;or\;straw\left(\%\right)\times 10^{-2}\right]$$ (3)

$$Total\;nitrogen\;uptake\left(kg\;ha^{-1}\right)=Nitrogen\;uptake\;grain+Nitrogen\;uptake\;straw$$ (4)

1. *Agronomic nitrogen use efficiency (ANUE,* kg kg^ − 1^*) is* the economic production obtained per unit of nitrogen applied. According to [59],

$$ANUE\;is\;calculated\;as\;\frac{Gf\left(kg\right)-Gu\left(kg\right)}{qNa\left(kg\right)},$$ (5)

where ***G*f** is the grain yield of the fertilized plot, ***G*u** is the grain yield in the unfertilized plot and **q*N*a** is the quantity of *N* applied.

1. *Physiological nitrogen use efficiency (PNUE,* kg kg^-1^) is the biological production per unit of nitrogen absorbed. It was calculated using the formula;

$$PNUE=\frac{Byf\left(kg\right)-Byu\left(kg\right)}{Nf\left(kg\right)-Nu\left(Kg\right)},$$ (6)

adopted from [60], where **B*yf*** is the biological yield (grain plus straw) of the fertilized plot (kg), **B*yu*** is the biological yield of the unfertilized plot (kg), **N*f*** is the total N uptake of the fertilized plot (kg), and **Nu** is the total N uptake of the unfertilized plot (kg).

1. Agro-physiological nitrogen use efficiency (APNUE, kg kg^-1^) is a measure of how efficiently the plant converts the applied nitrogen into increased biomass or yield.

$$APNUE=\frac{Gf\left(kg\right)-Gu\left(kg\right)}{Nf\left(kg\right)-Nu\left(kg\right)},$$ (7)

where **G*f*** is the grain yield of the fertilized plot (kg), *G****u*** is the grain yield of the unfertilized plot (kg), **N*f*** is the total N uptake of the fertilized plot (kg), and **Nu** is the total N uptake of the unfertilized plot (kg).

1. *Apparent recovery efficiency of nitrogen (AREN, %) is the* quantity of nitrogen absorbed per unit of nitrogen applied.

$$AREN=\frac{Nf\left(kg\right)-Nu\left(kg\right)}{qNa\left(kg\right)}\times 100,$$ (8)

adopted from [61], where **N*f*** is the total N uptake of the fertilized plot (kg), and **Nu** is the total N uptake of the unfertilized plot (kg)and **q*N*a** is the quantity of *N* applied (kg).

1. *Nitrogen use efficiency (NUE,* kg kg^-1^) measures the overall efficiency of nitrogen use by the plant [62].

$$NUE=PNUE\times AREN,which\;is\;simplified\;as\;\frac{Byf\left(kg\right)-Byu\left(kg\right)}{qNa\left(kg\right)}.$$ (9)

Where **B*yf*** is the biological yield (grain plus straw) of the fertilized plot (kg), **B*yu*** is the biological yield of the unfertilized plot (kg) and **q*N*a** is the quantity of *N* applied (kg).

1. *The nitrogen harvest index (NHI, %)* was determined as the ratio of nitrogen uptake by grain to nitrogen uptake by grain plus straw multiplied by 100, as described by [58].

### Statistical data analysis

The data collected in this experiment were collected following standardized protocols and the analysis was carried out using the SAS version 9.0 software computer program’s general linear model (GLM) procedure [63]. As described in Montgomery [64], the residuals were examined to verify the normal distribution and homogeneous variance model assumptions on the error terms for each response variable. Because the six treatment combinations were randomized within each block, the independence assumption was valid. When a treatment effect was significant, multiple means comparisons were performed at the 5% level of significance using the least significant difference (Fisher’s LSD) method to generate letter groupings.

### Partial budget analysis

A partial budget analysis was conducted to assess the economic viability of different treatments involving brewery sludge and blended NPS fertilizer. The analysis included partial budgeting, dominance analysis, and marginal analysis techniques. Partial budgeting and marginal analysis were employed to evaluate the economic aspects of the data collected. Partial budgeting serves as a tool to organize experimental data and provide insights into the costs and benefits associated with various treatment options. Meanwhile, marginal analysis is utilized to compare variable costs with the net benefits in order to determine the most cost-effective technology for recommendations derived from the experiment. Market prices for maize were obtained from local market rates in Ethiopian Birr (ETB) per kilogram at harvest time. Fixed prices for blended NPS fertilizer, BS as well as their transportation and application costs, were considered during the time of sowing.

The average partial budget for the six treatments was calculated based on income and expenses related to variable costs (Table 7). Net benefits were determined by subtracting total variable costs from gross field benefits, which were calculated by multiplying the farm price of the crop for each treatment. Variable costs excluded expenses for other agronomic practices like seed, land preparation, sowing, weeding, farm maintenance, and harvesting, as these costs were consistent across all treatments. The sum of variable costs was deducted from gross field benefits to obtain net benefits. The average yield was adjusted downwards by 10% to account for differences between experimental field conditions and expected yields under farmers’ practices within the same treatments. Based on the results of the dominance analysis, treatments were chosen in increasing order of total variable costs. For each pair of ranked treatments, the percent marginal rate of return (MRR) was calculated. The MRR (%) between any pair of non-dominated treatments was the return per unit of investment in fertilizer. It was calculated by dividing the change in net benefit by the change in variable costs. Analysis of the marginal rate of return (MRR) was carried out for non-dominated treatments, and the MRRs were compared to the minimum acceptable rate of return (MARR) of 100% to determine the optimum treatment [65].

**Table 7:** Partial budget analysis for maize yield as influenced by brewery sludge and blended NPS fertilizer rates for maize crop production.

Treatment	UAGY (kg ha^-1^)	AGY (kg ha^-1^)	GFB (ETB ha^-1^)	TVC (ETB ha^-1^)	NB (ETB ha^-1^)	MRR (%)
Control	6132.6	5519.34	99348.12	0	99348.12	–
100% RDBS	7773.7	6996.33	125933.94	703	125230.94	3681.77
75% RDBS + 25% RDNPS	10620.6	9558.54	172053.72	1065.75	170987.97	12613.93
50% RDBS + 50% RDNPS	8439.2	7595.28	136715.04	1428.5	135286.54	D
25% RDBS + 75% RDNPS	7549.0	6794.1	122293.8	1791.25	120502.55	D
100% RDNPS	6393.5	5754.15	103574.7	2154	101420.7	D

**BS** =  brewery sludge; **ETB** =  Ethiopian Birr; **UAGY** = unadjusted Grain Yield; **AGY** = Adjusted Grain Yield; **GFB** =  Gross field benefit; **TVC** =  Total variable cost; **NB** = Net benefit; local price of maize =  18ETB kg^-1^; costs of blended NPS fertilizer =  19.04 ETB kg^-1^; transportation cost of NPS =  130 and its labor cost for application = 120 ETB/8hrs/100 kg blended NPS fertilizer; costs of brewery sludge =  60ETB t^-1^; transportation of brewery sludge =  60 ETB and application cost =  43 ETB/2 hrs and 45 minutes for 10 ton brewery sludge

## Results and discussion

### Physicochemical properties of soil and brewery sludge

The findings from the analysis of the soil and brewery sludge are detailed in Table 2. The soil analysis revealed that the Kudmi soil belongs to the clay textural class and has a high cation exchange capacity of 30 cmol (+)/kg. The pH of the soil in the study area was 5.32, which is moderately acidic with moderate total nitrogen (0.28%), available phosphorus (0.398 mmol/L [67] and organic matter (2.42%) [68]. The BS exhibited a high pH value of 6.69, suggesting the potential to raise the low soil pH of 5.32 to slightly acidic upon application (Table 2). Furthermore, the available phosphorus (AP) and total nitrogen (TN) content of BS was higher than that of the soil, as the sludge involves the use of grains and other organic materials rich in nutrients such as phosphorus and nitrogen. Additionally, its cation exchange capacity (CEC) value was higher than that of the soil, likely due to its highly porous structure with a large surface area, providing more sites for cation exchange to occur [70]. This increased surface area allows for greater interaction between the BS and soil particles, leading to higher CEC values [71].

**Table 2:** Physicochemical properties of the soil and BS before sowing maize.

Parameter	Soil	BS	References
Sand	17	--	
Silt	28	--	
Clay	55	--	
Texture class	Clay	--	USDA [66]
pH	5.32	6.69	Tadesse *et al* [67]
Organic matter (%)	3.97	13.9	Landon [68]
Total *N* (%)	0.28	4.23	Tadesse *et al* [67]
Av. P (mmol/L)	0.398	1756.7	Olsen [54]
CEC [cmol(+)/kg soil]	30	40.0	Hazelton and Murphy [69]

### Maize yield and yield component parameters

#### Aboveground dry biomass yield.

The co-application of BS and blended NPS fertilizer had a highly significant (P < 0.001) effect on the aboveground dry biomass yield of maize. The combined application of 25% RDNPS +  75% RDBS resulted in the highest aboveground dry biomass yield (20791.5 kg ha^-1^), an increase of 48.96% compared with that of the control treatment (Table 3). An increase in aboveground dry biomass yield could result from the overall improvement in vegetative growth as a result of the combined application of BS and blended NPS fertilizers. Furthermore, the increase in the aboveground dry biomass yield of maize from plots fertilized with combined BS and blended NPS fertilizer could be attributed to the plants receiving a proper and balanced supply of nutrients throughout the growth period. The observed similarity in values between the control treatment and 100% RDNPS may indicate that nitrogen application rates were insufficient to elicit significant differences, suggesting that environmental factors and soil type could have influenced nitrogen uptake and maize yield outcomes [72]. These findings are in accordance with those of Fashaho [73], who reported that the combined application of 10 t ha^-1^ bioslurry with 90 kg ha^-1^ mineral N increased the aboveground dry biomass yield of maize by 3.3 times compared with that in the control treatment. Similarly, a study on bioslurry and chemical fertilizer found that a combination of 25% bioslurry and 75% RDNPS fertilizer produced the highest (24.40 t ha^-1^) maize biomass yields in Hawassa, Ethiopia [74]. These findings were also consistent with those of [75], who reported that combining organic and mineral fertilizers increased grain weight, grain and straw yield and biological yield. Similarly, [76] reported that the combined application of inorganic and organic fertilizers resulted in greater total dry matter in maize than did the application of either fertilizer source alone.

**Table 3:** The effects of sole and integrated application of brewery sludge and blended NPS fertilizer on the above-ground biological yield, grain yield, straw yield, and harvest index of maize.

Treatment	AGDBY(kg ha^-1^)	GY (kg ha^-1^)	SY (kg ha^-1^)	HI (%)
Control	13957.6c	6132.6c	7825.0b	43.73b
100% RDNPS	14298.7c	6393.5c	7905.2b	44.65b
75% RDNPS + 25% RDBS	15462.8bc	7549.0b	7913.9b	48.82a
50% RDNPS + 50% RDBS	16877.9b	8439.2b	8438.6b	50.03a
25% RDNPS + 75% RDBS	20791.5a	10620.6a	10171.0a	51.09a
100% RDBS	15717.1bc	7773.7b	7943.4b	49.57a
LSD (0.05)	1806.6^***^	1140.8^***^	891.6**	3.96**
CV (%)	6.14	8.02	5.86	3.93

Means in columns with the same letter(s) are not significantly different at the 5% level of significance; **LSD** =  least significant difference; *=significant at P ≤  0.05;

*** =  highly significant at P ≤  0.001; **CV (%)** =  coefficient of variation; **GY**=grain yield; **SY**=stover yield; **AGDBY**=above ground dry biological yield; and **HI**=harvest index

#### Grain yield.

The grain yield of maize was significantly affected (P < 0.001) by the combined application of BS and NPS fertilizer. The highest grain yield (10620.6 kg ha^-1^) was recorded in the combined application of 25% RDNPS +  75% RDBS, with an increase of 73.2% compared with that in the control (untreated) treatment (Table 3). This increased grain yield of maize might be due to sufficient nutrient retention and availability for crops, increased kernels per ear and thousand kernels weight, all of which resulted in improved maize yield [77]. This increase in maize grain yield may also be due to synergistic effects of nutrient interactions that improve soil structure [78,79], water holding capacity [80], microbial activity, as well as the fertilizer nutrient utilization rate of soil [81]. Consistent with the results of this study, [73] also reported that the application of 10 t ha^-1^ bioslurry along with 90 kg ha^-1^ mineral N increased the grain yield by 3.08 times over that in the control treatment. In agreement with these findings, the combined application of 4 t FYM with 75/60 kg N/P ha^-1^ is an inexpensive and beneficial combination for increasing hybrid maize (BH140) production in the West Hararghe zone of Eastern Ethiopia [82]. Tuyishime [83] also reported an increase in maize grain yield in northern Rwanda by combining 10 t ha^-1^ bioslurry and 50 kg ha^-1^ urea.

#### Stover yield.

Analysis of variance indicated that the stover yield of maize was high significantly (P < 0.01) affected by the combined application of BS and blended NPS fertilizer. The highest stover yield of maize (10171 kg ha^-1^) was recorded in plots treated with 25% RDNPS + 75% RDBS, whereas the lowest (7825 kg ha^-1^) was from the untreated (control) plot. This might be due to the role of BS in improving soil structure, water retention and nutrient availability. The organic matter in the sludge enhances soil fertility, microbial activity and nutrient cycling, supporting better maize growth and stover production. In agreement with our finding, Tsehay *et al* [74] found and reported that the combined application of 25% bio-slurry and 75% chemical fertilizer resulted in the highest stover yield of maize (11.5 t ha^ − 1^), followed by 50% bio-slurry and 50% chemical fertilizer and 100% bio-slurry, which are 11.3 and 10.17 t ha^ − 1^, respectively. The lowest stover yield (8.43 t ha^ − 1^) was recorded in the control group. Similarly, Tukur and Kesarwani [84] and Yigermal *et al* [85] reported that an application of 5 t ha^ − 1^ of the poultry manure with 50% RDF (50 N +  30 P +  20 K +  10 Z kg ha^ − 1^) produced the highest (13.843 t ha^ − 1^) stover yield, whereas an application of 100% RDF (100 N +  60 P +  40 K +  20 Z kg ha^ − 1^) produced the lowest (8.321 t ha^ − 1^) stover yield.

#### Harvest index.

Analysis of variance indicated that the harvest index was high significantly affected (P < 0.01) by the combined application of BS and blended NPS fertilizers. The highest harvest index (51.09%) was recorded in the application of the 25% RDNPS + 75% RDBS treatment, which was significantly at par with the application of the rest combinations ratio of RDBS with RDNPS, whereas the lowest (43.73%) was recorded in the untreated or control treatment, followed by the sole RDNPS application (Table 3). These differences could be attributed to the increased availability and uptake of essential nutrients from the applied BS and blended NPS fertilizers and sink to the grain yield of maize. Sole application of RDNPS also yielded lower results, indicating that the synergistic effect of combining organic and inorganic fertilizers is crucial for maximizing maize productivity [45]. In agreement with the findings of this study, those of Muhmood *et al* [86] revealed that the addition of 0.6 t ha^-1^ bio slurry with 50% recommended N had the highest (16%) harvesting index of okra, whereas the lowest (11.9%) was from the control treatment. Similarly, Fashaho [73] reported that the highest harvest index (35.1%) of maize was recorded after the application of 10 t ha^-1^ bio slurry fertilizer in combination with 30 kg ha^-1^ mineral nitrogen fertilizer, while the lowest was recorded after the sole application of 15 t ha^-1^ bio slurry organic fertilizer at high altitudes in the Gicumbi district, northwestern Rwanda. The results of this study, however, contradict the findings of Ahmad *et al* [87], who reported that the integrated application of organic, inorganic and biofertilizers had a non-significant effect on the harvest index of maize in low-fertile soil.

### Grain and stover nitrogen concentration of maize

#### Percentage of Nitrogen concentration in Grain.

Grain nitrogen concentration was significantly (P < 0.05) affected by the combined application of BS and blended NPS fertilizer as soil fertility amendments. The highest (1.71%) grain nitrogen concentration percentage was found following the application of 25% RDNPS + 75% RDBS, whereas the lowest (1.32%) was from the untreated treatment (Table 4). The grain N concentration progressively improved as the amount of BS increased and the amount of blended NPS fertilizer decreased. This is related to the fate of nitrogen from blended NPS fertilizer, which leaches. However, the combination of blended NPS fertilizer and BS may affect nitrogen release dynamics in the soil because blended NPS fertilizer is a readily available source of nitrogen, but BS can behave as a slow-release source over time. This combination has the ability to provide a consistent supply of nitrogen to crops, resulting in higher nitrogen concentrations. In agreement with this study, Hossaen *et al* [88] reported that lower N concentration on grain of rice was found with a ratio of 75% urea: 25% organic sources, while maximum N concentration was obtained with 25% urea: 75% organic sources. According to Yigermal *et al* [85], the maximum total N after each crop harvest was also found in the treatment where organic fertilizers were applied, integrated with low mineral N sources fertilizer. Similarly, the maximum (1.43%) N concentration in grain maize was found in the treatment receiving 75% N from mineral sources and the remaining 25% from FYM, followed by 1.39% in the treatment receiving N in a 50:50 ratio [89].

**Table 4:** Effects of the sole and integrated brewery sludge and blended NPS fertilizer on the percentage of nitrogen concentration in the grain and stover of maize.

Treatment	PNCG (%)	PNCS (%)
Control	1.32c	0.62c
100% RDNPS	1.33c	0.63c
75% RDNPS + 25% RDBS	1.58ab	0.84ab
50% RDNPS + 50% RDBS	1.44bc	0.79bc
25% RDNPS + 75% RDBS	1.71a	1.00a
100% RDBS	1.48b	0.86ab
LSD (0.05)	0.14^***^	0.19**
CV (%)	5.29	13.29

Means in columns with the same letter(s) are not significantly different at the 5% level of significance; **PNCG** = percentage of nitrogen concentration in the grain; **PNCS** = percentage of nitrogen concentration in stover; **LSD** =  least significant difference;

*** =  very highly significant at P ≤  0.001; CV (%) =  coefficient of variation

#### Percentage of nitrogen concentration in stover.

Stover nitrogen concentration was highly and significantly (P < 0.01) affected by the combined application of BS and blended NPS fertilizer. The highest (1.00%) stover nitrogen concentration percentage was found with the application of 25% RDNPS + 75% RDBS, whereas the lowest (0.62%) was recorded in the control treatment (Table 4). This might be due to the combination of blended NPS fertilizer and BS providing a balanced and readily available supply of nitrogen to the plants, leading to increased nitrogen uptake and accumulation in the stover [90]. In harmony with this finding, Rehim *et al* [91] found that the highest (0.59%) N concentration in straw of wheat was recorded with the combined application of 75% urea +  25% FYM, while the lowest (0.29%) was found with no/any fertilizer application. Similarly, Amanullah *et al* [92] and Shah and Ahmad [93] also reported that the highest nitrogen concentration in wheat straw was recorded in the combined application of organic manure and mineral fertilizer.

### Grain, stover and total nitrogen uptake of maize

#### Grain nitrogen uptake.

The combined application of BS and blended NPS fertilizer had a very highly significant (P < 0.001) effect on maize grain nitrogen uptake. The application of 25% RDNPS + 75% RDBS resulted in the highest (181.72 kg ha^-1^) grain nitrogen uptake, whereas the untreated plot had the lowest (81.65 kg ha^-1^) (Table 5). This might be due to the improvement of soil fertility and biological activity with BS, which in turn affects nitrogen availability for crops as reported by [94,95]. The present results are consistent with Khan *et al* [89] the maximum N uptake of 49.72 kg ha^-1^ was obtained in treatment receiving 75% N from urea and 25% from FYM.

**Table 5:** Grain nitrogen uptake, stover nitrogen uptake and total nitrogen uptake of maize affected by the sole and integrated application of brewery sludge and blended NPS fertilizer.

Treatment	GNUP (kg ha^-1^)	SNUP (kg ha^-1^)	TNUP (kg ha^-1^)
Control	81.83c	48.70b	130.53c
100% RDNPS	85.57c	50.40b	135.97c
75% RDNPS + 25% RDBS	119.50b	66.86b	186.36b
50% RDNPS + 50% RDBS	121.88b	67.45b	189.34b
25% RDNPS + 75% RDBS	181.72a	102.28a	284.00a
100% RDBS	114.93b	69.26b	184.19b
LSD (0.05)	21.73^***^	22.2**	28.53^***^
CV (%)	10.16	18.08	8.47

Means in columns with the same letter(s) are not significantly different at the 5% level of significance; **GNUP** = grain nitrogen uptake; **SNUP** = stover nitrogen uptake; **TNUP** = total nitrogen uptake; **LSD** =  least significant difference;

*** =  very highly significant at P ≤  0.001; *= significant at p ≤ 0.05 **CV (%)** =  coefficient of variance

#### Stover nitrogen uptake.

The analysis of variance (ANOVA) revealed that the stover nitrogen uptake of maize was high significantly (P < 0.01) affected by the combined application of organic BS and blended NPS fertilizers. The highest nitrogen uptake (102.28 kg ha^-1^) in stover of maize was recorded with the application of 25% RDNPS + 75% RDBS, whereas the untreated plots had the lowest (48.70 kg ha^-1^) nitrogen uptake by stover of maize, as shown in Table 5 below. This might be due to the combined application of BS and blended NPS fertilizer, which enhances soil pH and organic matter, leading to better nutrient retention and availability to the crop [96]. In agreement to this study Baradhan *et al* [97], conducted a field experiment in the Experimental Farm of Annamalai University and reported that the combined application of 100% recommended dose of inorganic fertilizer (RDF) with 2.5 t ha^-1^ poultry manure resulted in the highest nitrogen availability and uptake by maize crop.

#### Total nitrogen uptake.

The ANOVA revealed that the stover nitrogen uptake of maize was very highly and significantly (P < 0.001) affected by the combined application of organic BS and blended NPS fertilizers. The highest total nitrogen uptake(284 kg ha^-1^) of maize was recorded with the application of 25% RDNPS + 75% RDBS, whereas the untreated plots had the lowest (130.53 kg ha^-1^) total nitrogen uptake by maize as shown in Table 5 below. Similar to this result, Khan *et al* [89] reported that the application of 25% FYM + 75% N resulted in the highest total nitrogen uptake and the lowest was recorded from the untreated plot of maize crop. Our result is in corroborated the findings of Dunjana *et al* [98], Negassa *et al* [99] and Rusinamhodzi *et al* [100], those stated that the integrated application of organic and mineral fertilizers at appropriate rates can be an effective approach to improve maize N uptake.

### Nutrient use efficiency

Nutrient Use Efficiency (NUE) can be greatly influenced by the management of fertilizers, soil, and irrigation [101]. With a rising need for fertilizer nutrients to support global food demands, the limited availability of fertilizer resources and concerns about their environmental impacts have heightened. Consequently, there is a growing emphasis on enhancing NUE without compromising crop productivity. It measures how effectively crops absorb and utilize nutrients to achieve optimal yields. As such, the NUE concept encompasses three key plant processes: nutrient uptake, assimilation, and utilization [102,103]. It can be also characterized using several indices [104].

#### Agronomical nitrogen use efficiency.

Agronomic nitrogen use efficiency (ANUE) is a metric that includes yield potential of an applied fertilizer and relates directly to economic return [105]. According to the results of ANOVA, the combined application of BS and blended NPS fertilizer had not statistically significant (P > 0.05) effect on the ANUE of maize. However, the highest ANUE (13.94 kg kg^-1^) was recorded with the application of 25% RDNPS + 75% RDBS followed by the application of 100% RDNPS (13.73 kg kg^-1^), whereas the lowest ANUE (3.88 kg kg^-1^) was obtained from the application of 100% RDBS (S1 Fig). The highest agronomic nitrogen use efficiency observed could be attributed to the synergistic effects, balanced nutrient composition, and enhanced soil health resulting from the combined use of BS and blended NPS fertilizer. These results are in line with those of El-Syed *et al* [106] compost with inorganic NPK did not show any significant effect on ANUE compared to adding inorganic NPK alone. In the same way, Asaye *et al* [107], reported that the integrated application of vermicompost with blended NPS fertilizer resulted in a higher ANUE of maize than the application of sole blended NPS fertilizer. Similarly, Dorsey [108] and Ramesh [109] concluded that the combined application of vermicompost with urea fertilizer produced higher agronomic nitrogen use efficiency of the wheat crop.

#### Physiological nitrogen use efficiency.

Physiological nitrogen use efficiency (PNUE) represents the fraction of plant acquired N that is converted to grain yield. The ANOVA revealed that PNUE of maize was significantly (P < 0.05) affected by the combined application of BS and blended NPS fertilizer. The highest PNUE (66.71 kg kg^-1^) was recorded with the sole application of 100% RDNPS, whereas the lowest PNUE (27.69 kg kg^-1^) was obtained with the combined application of 75% RDNPS with 25% RDBS as shown below in S1 Fig. This result may be due to the application of 100% RDNPS, which likely provided the maize plants with optimal levels of readily available nitrogen, phosphorus and sulfur, which are essential nutrients for plant growth and development. In agreement with this finding, Bekalo [110] reported that application of the recommended dose of NP fertilizer increased PNUE between 39.6 and 60.6% against the combined application of NP fertilizer with FYM and sole FYM in potato crops. However, our result was in contrast with those of Agegnehu *et al* [7] who reported that the application of biochar +  23 kg N ha^-1^ resulted in the highest physiological nitrogen use efficiency of 33 kg kg^-1^ N at Holetta and 48 kg kg^-1^ N at Robgebeya.

#### Agro-physiological nitrogen use efficiency.

According to the ANOVA, the combined application of BS and blended NPS fertilizer had a non-significant (P > 0.05) effect on the agro-physiological nitrogen use efficiency (APNUE) of maize. However, the highest APNUE (37.78 kg kg^-1^) was found with the sole application of 100% RDNPS, whereas the lowest APNUE (25.46 kg kg^-1^) was recorded from the combined application of 75% RDNPS + 25% BS (S1 Fig). This result might be due to the nutrient balance, enhanced nutrient interactions, organic matter content and slow release of nutrients, which leads to higher APNUE maize. In line with the report of this study, Fazily *et al* [111] conducted research at the Agronomy Research Farm of Haryana Agricultural University under irrigation and reported that the highest APNUE (54.17 kg kg^-1^) of maize was recorded under the application of 25% RDN + 75% FYM followed by 50% RDN + 50% FYM (50.70 kg kg^-1^), whereas the lowest APNUE (47.54 kg kg^-1^) was observed with the application of 100% RDN +  25% FYM in comparing the combined application of chemical fertilizer with farm manure only.

#### Apparent recovery efficiency of nitrogen.

Analysis of data showed that the apparent recovery efficiency of nitrogen (AREN) of maize was significantly (P < 0.05) affected by the combined application of BS and blended NPS fertilizer (S1 Fig). The highest AREN (47.67%) was found with the combined application of 25% RDNPS + 75% RDBS followed and statistically at par with the combined application of 75%RDNPS + 25%RDBS (46.53%), whereas the lowest AREN (12.69%) was found in plots treated with only 100% RDBS. This could be due to the combined application of BS and blended NPS, which may enhance nutrient availability, promote microbial activity, and improve soil structure, leading to higher AREN. Our result was in consistent with those of Agegnehu *et al* [7] those reported that the AREN responded significantly to biochar with N fertilizer.

#### Utilization efficiency of nitrogen.

The utilization efficiency of nitrogen (UEN) for a cereal crop depends on its ability to acquire nitrogen from the available supply and how effectively it utilizes nitrogen to produce grain [7,112,113]. In most cases, annual crops only take up nitrogen from the soil at optimal fertilizer rates for a period of 8–12 weeks, leading to a mismatch between nitrogen availability and crop demand, which contributes significantly to nitrogen losses [114]. The result of this study indicated that the combined application of BS and NPS fertilizer did not have a significant effect (P > 0.05) on the nitrogen utilization efficiency of maize. However, the highest UEN (21.22%) was observed when 25% RDNPS with 75% RDBS was applied, while the lowest UEN (4.16%) was recorded with the sole application of 100% RDBS (10 t ha^-1^ BS), as shown in Table 6. This discrepancy may be due to the tendency of nitrogen in organic amendments to undergo immobilization, resulting in plants recovering limited amounts of nitrogen from these amendments in the short term [7]. However, split applications of urea, particularly three-way splits (one-fourth at sowing, one-half at vegetative stages, and one-fourth at tasseling) compared to single applications, enhance the UEN of maize [72,115].

**Table 6:** Utilization Efficiency of Nitrogen (%) and Nitrogen harvest index of maize affected by the combined application of brewery sludge and blended NPS fertilizer.

Treatment	UEN (%)	NHI
Control	--	62.43
100% RDNPS	17.96a	62.81
75% RDNPS + 25% RDBS	12.54ab	64.90
50% RDNPS + 50% RDBS	13.21ab	64.50
25% RDNPS + 75% RDBS	21.22a	64.31
100% RDBS	4.16b	63.02
LSD (0.05)	12.27^NS^	8.57^NS^
CV (%)	47.16	7.40

Means in columns with the same letter(s) are not significantly different at the 5% level of significance; **UEN** = utilization efficiency of Nitrogen; **NHI**=nitrogen harvest index; **LSD** =  least significant difference; **NS** =  non-significant at P >  0.05; **CV (%)** =  coefficient of variance

#### Nitrogen harvest index.

Nitrogen harvest index (NHI) indicates the level of efficiency of plants to use acquired nitrogen for grain formation. A high NHI indicates efficient utilization of nitrogen. The analysis results of this study showed that NHI was not significantly (P > 0.05) affected by the combined application of BS and blended NPS fertilizer. This might be due to the brewery sludge not having provided sufficient readily available nitrogen to enhance the NHI, as its organic matter content can lead to slower nitrogen release compared to synthetic fertilizers [116].

This result is in line with Agegnehu [117], who found and report that NHI of barley was not significantly affected by the application of organic amendments and chemical nitrogen fertilizer at Holotta, Ethiopia.

### Partial budget analysis

According to the manual for the economic analysis of International Maize and Wheat Improvement Centre (CIMMYT [65]), combined applications of 75% RDBS with 25% RDNPS fertilizer rates resulted in the highest net benefit of 170987.97 ETB ha^-1^ with the most acceptable MRR (12613.93%), followed by the sole application of 100% recommended brewery sludge (10 t ha^-1^) resulted in a net benefit of 125230.94 ETB ha^-1^ with 3681.77% MRR whereas the lowest from the control (Table 7). As a result, this treatment combination was the most recommended and cost-effective for maize production. This implies that using the best organic and inorganic fertilizers is critical for improving soil fertility and crop productivity. The result was in line with Jinwei and Lianren [118] and Lingaraju *et al* [119] that a combined application of organic manure and inorganic fertilizer produced a high net benefit income and cost-benefit ratio compared with the sole application of either organic or inorganic fertilizer on maize crops.

## Conclusion and recommendations

The results of a field experiment conducted in the North Mecha district of Northwestern Ethiopia show that using both BS and blended NPS fertilizers, either alone or in combination, has considerable potential to increase maize output in the research area. The results clearly show that the application of 25% RDNPS with 75% RDBS fertilizer ratios led to the maximum maize above-ground biological yield, grain yield, nitrogen uptake and overall nitrogen use efficiency when compared to sole chemical fertilizer applications or no fertilizer use. The study emphasizes the need to address soil fertility issues in Ethiopia through targeted fertilizer applications to boost maize productivity. Farmers in the North Mecha district can use the determined optimal fertilizer combination as a realistic and successful technique to maximize maize yields and economic returns. However, further research is required to fine-tune the rates and application methods of BS, NPS and their combination in order to achieve long-term agronomic gains in maize production. Long-term success in increasing maize yield in the region will depend on continuous monitoring and adaptation of fertilizer techniques depending on local soil conditions and crop requirements.

## Supporting information

S1 Fig Nitrogen use efficiency indices of maize as affected by sole and integrated brewery sludge and blended NPS fertilizer. (DOCX)

S1 Data PLOS ONE raw data. (ODS)
