# Supplementary material for: From brewery waste to agricultural wealth: Enhancing nitrogen use efficiency and productivity of maize through brewery sludge and blended NPS fertilizer in North Mecha District, Northwestern Ethiopia
Source: PLoS One. 2025 May 8;20(5):e0319958. doi: 10.1371/journal.pone.0319958 (PMC12061191; doi:10.1371/journal.pone.0319958)
Supplement: S1 Fig — (DOCX) [file pone.0319958.s001.docx]

Sl Fig: Nitrogen use efficiency indices of maize as affected by sole and integrated brewery sludge and blended NPS fertilizer
